# Low Magnetic Field Detection Using a CuPt Nano Structure Made on a SiO_2_/Si Structure

**DOI:** 10.3390/s91209734

**Published:** 2009-12-02

**Authors:** Hassan Hajghassem, Seyedeh Maryam Banihashemian, Majidreza Aliahmadi

**Affiliations:** 1 Electrical Engineering Department Shahid Beheshti University, Tehran, Iran; 2 Department of Physics Islamic Azad University Qom branch, Qom Payam Noor University, Iran; 3 Electronic Research Center, Tehran, Iran

**Keywords:** Cu-Pt nano clusters, I-V Curve, magnetic field detection

## Abstract

A Si/SiO_2_/CuPt structure is formed by depositing a very thin SiO_2_ layer between CuPt and P-type Si layers using e-beam evaporation. SEM images show the formation of CuPt nano clusters with an average size of less than 100 nm. This structure shows high sensitivity to applied magnetic fields at 77K and at low and high dc voltages such that magnetic field as low as 6 mT is detected using I-V and I–B measurements. The variation of current with various magnetic field strength at the constant voltage shows also an oscillatory behavior. The sensitivity of this structure to magnetic fields is believed to be due to small nano size of the platinum–copper structures as well as their discrete energy states and the tunneling of carriers into the insulating layer. Our results indicate that this structure may be a good candidate for small, simple, low cost and sensitive low magnetic field detectors.

## 1. Introduction

Mixed magnetic sensors provide a very sensitive measurement of low fields [1]. Currently, commonly used cryogenic magnetic sensors are based on the Hall Effect, or the magneto resistive (MR) effect in semiconductors. However, both of these types of sensors are expensive. The most desirable operating temperature range for cryogenic magnetic field sensor is at around the liquid nitrogen temperature [2]. Kvon [3] and Baturina [4] have investigated the variation in magnetic field at temperature close to 0K in platinum silicide superconductors. A magnetic field sensor made of magnetic tunnel junction of ferromagnetic/ insulator (FM/I) layers was reported in 2006. Magnetic tunnel junctions (MTJs) consisted of two ferromagnetic metal layers separated by a thin insulating oxide barrier [5-7]. When a thin insulating layer is placed between two metal or superconductor layers and if other conditions are set, the tunneling of carriers through the insulating layer may take place. As a result, the wave function of carriers extends into the insulating layer and the probability of existence of carriers on the other side of the insulating layer increases. By solving the wave function relation on two sides of insulating layer and based on quantum mechanical equations, one can theoretically calculate this current [8]. Schoonus has investigated the variation of current-voltage due to magnetic field in Al/SiO_2_/Si:B structures. According to his report, this structure is sensitive to a magnetic field of 500 mT at 4K [9]. The variation of magnetic resistance in CuPt/SiO_2_/Si structure has been recently investigated and reported by our team [10].

**Figure 1.**
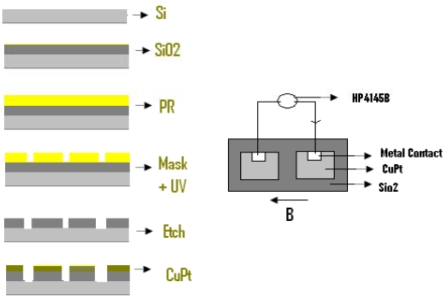
Cu-Pt film (8 nm) on a p type silicon with an ultra thin insulating (SiO_2_) layer and CuPt (8nm)/SiO_2_ (5 nm)/Si (50,000 nm)/SiO_2_ (5 nm)/CuPt (8 nm) array structure Fabrication procedure is shown on the left and the measurement procedure is shown on the right.

In this work we have shown that a very low magnetic field can be sensed using nonmagnetic material of nano clusters of CuPt on SiO_2_/Si using a CuPt (8 nm)/SiO_2_ (5 nm)/Si (50,000 nm)/SiO_2_ (5 nm)/CuPt (8 nm) atructure at liquid nitrogen temperature (as shown in Figure 1). This structure shows high sensitivity to applied magnetic fields at 77K and at low and high dc voltages.

I-V measurement in the Si/SiO_2_/CuPt structure is made at 77 K and 300 K respectively. All measurements are made in the presence and absence of magnetic fields applied in parallel to the formed junction. Magnetic fields as low as 6 mT are detected using I-V and I–B measurements made in the Si/SiO_2_/CuPt structure at 77 K. The current as a function of magnetic field has an oscillatory behavior. This curve has the same oscillatory behavior for other voltages with maximum variation occurring near the origin (*i.e.*, zero magnetic fields) SEM images show the formation of CuPt nano clusters with an average size of less than 100 nm. The magnetic behavior of these nanosized metals is a new observation that will be explained below and is believed to be due to the small nanosize of the platinum and copper structures as well as their discrete energy states and the tunneling of carriers into the insulating layer.

## 2. Results and Discussion

**Figure 2.**
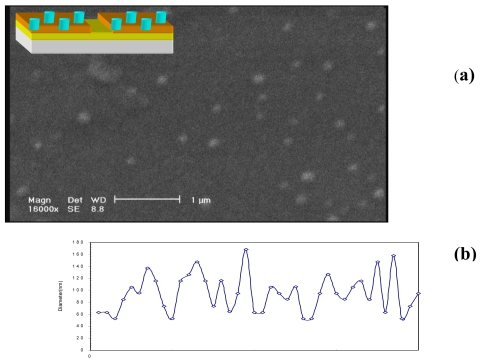
(a) SEM image of CuPt nano cluster on SiO2/Si surface. (b) Distribution of cluster diameter in an area of 5 μm by 6 μm.

In this work a very thin SiO_2_ layer is placed between CuPt and Silicon and CuPt/SiO_2_/Si/SiO_2_/CuPt structure is formed. Figure 2a shows the SEM images of the nanocluster CuPt structure on the SiO_2_/Si. As it can be seen in this Figure that the CuPt clusters are randomly distributed on a 30 μm^2^ surface. The diameters of these clusters are less than 100 nm. Figure 2b shows the distribution of cluster size in an area of 5 μm by 6 μm.

**Figure 3.**
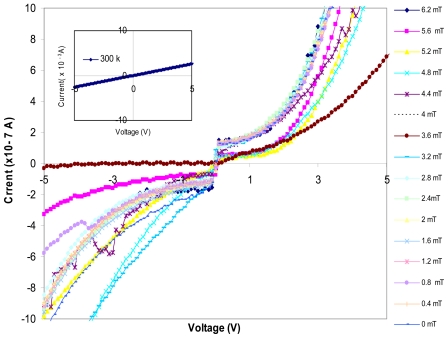
I-V Curve made at liquid nitrogen temperature and room temperature (inset of Figure3) for CuPt/Sio_2_/Si/Sio_2_/CuPt structure respectively. External magnetic field is applied parallel to the surface of the device for low voltages.

**Figure 4.**
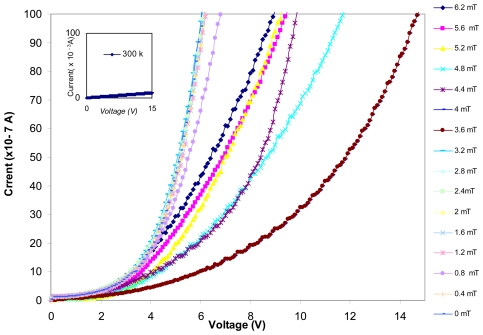
I-V Curve made at liquid nitrogen temperature and room temperature (inset of Figure 4) for CuPt/Sio_2_/Si/Sio_2_/CuPt structure respectively. External magnetic field is applied parallel to the surface of the device for higher voltages.

As it can be seen, the average diameter size of these clusters is 96 nm and their approximate thickness is 8 nm. The Current-Voltage characteristics of this structure at 77 K and 300 K is shown in Figures 3, 4 and their insets, respectively. Variation of the I-V curve due to the external field exposed in parallel to the junction is also investigated in this work. As it can be seen in Figures 3, 4 there are some variations in the I-V curve due to different magnetic field strengths applied at liquid nitrogen temperature. However, The I-V curve is ohmic at 300 K and has no response to an external field (inset of Figures 3, 4). As we can see in these Figures the I-V curves have barrier type behavior at 77 K, which indicates that there is a potential barrier between the CuPt and Si/SiO_2_ layers. However, at higher temperature close to room temperature the thermionic emissions, the field effect emissions and the tunneling emission in spot size contacts of CuPt structure on Si/SiO_2_, superimpose and an ohmic behavior is observed.

**Figure 5.**
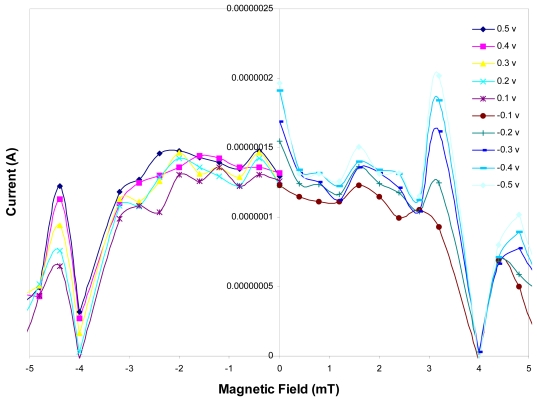
Current as a function of external field applied to the junction at 77 K for different voltages.

The slope of the curve at liquid nitrogen temperature varies by increasing the strength of the magnetic fields. The resulting current as a function of different magnetic field strength is also shown in Figure 5. As it is shown in this Figure, the variation of current with magnetic field has an oscillatory relation. Such that the envelope of the curve has almost a Gaussian shape with its peak at zero magnetic field. These curves are symmetric with respect to the direction of applied field and as we can see the current decreases by increasing the magnitude of the applied fields. Non-magnetic platinum atoms can exhibit magnetic behavior when bound together in a form of small clusters [9,11,12]. However, based on SEM images and I-V and I-B curves obtained in this work, the observed magnetic behavior as will be explained below is believed to be due to the small nanosize of the platinum and copper structure as well as their discrete energy states. Since the energy states in CuPt are discrete and by considering the fact that the tunneling of carriers into the insulating layer is only satisfied when the carriers tunnel from full occupied states into the empty states of the other side, this magnetic behavior varies with the variation in voltage and consequently variation in Fermi energy states.

However, by applying external magnetic field there will be a change in carrier concentration and as a result change in tunneling current and slope of the I-V curve. The magnetic sensitivity observed in this structure indicates the potential of CuPt nano clusters for sensing magnetic field as low as the earth magnetic fields.

## 3. Experimental Section

P-type Si doped with 7 × 10 ^14^ Boron atom/cm^3^ is used in this work. After RCA cleaning followed by plasma cleaning, 200 nm of silicon dioxide is deposited on the surface. Annealing for 30 minutes at 500 °C is performed after oxide deposition; Patterning with an array structure is formed as shown in Figure 1. A 1 mm × 1 mm window is opened on SiO_2_ layer using chemical etching such that a very thin SiO_2_ layer remained on the surface. Deposition of platinum and copper is performed on the surface, such that 8 nm of Cu-Pt is formed. Thirty minutes of annealing at 400 °C followed after the deposition. Molybdenum and aluminum (*i.e.*, 100 nm and 500 nm, respectively) are then deposited on top of the Cu-Pt layer using e-beam evaporation followed by 20 minutes of annealing at 200 °C. After preparation of the devices they are tested both with and without the presence of magnetic fields as shown in Figure 1. Scanning Electron Microscope SEM: XL30 Philips, HP4145B semiconductor parameter analyzer, Microstructure Measurements software and other computer software are used for measurements and analysis.

## 4. Conclusions

The CuPt/SiO_2_/Si/SiO_2_/CuPt structure presented in this work shows a good response to low magnetic field (*i.e.*, less than 6 mT) at 77 K, which is a new observation in this structure. The sensitivity of this structure to magnetic fields is believed to be due to small nano size of the Pt–Cu structures as well as their discrete energy states and the tunneling of carriers into the insulating layer. The variation of current due to external magnetic field has an oscillatory behavior such that the average current increases with the voltage. The variation of current with various magnetic field strength at the constant voltage shows also an oscillatory behavior. Since this structure is very sensitive to low intensity magnetic fields, it may also be used for measuring earth magnetic fields. Small size, simple processing fabrication, low cost and high sensitivity are some advantages of this structure to be considered as a magnetic field detector.
